# A dynamic multi‐scale occupancy model to estimate temporal dynamics and hierarchical habitat use for nomadic species

**DOI:** 10.1002/ece3.4822

**Published:** 2019-02-05

**Authors:** Adam W. Green, David C. Pavlacky, T. Luke George

**Affiliations:** ^1^ Bird Conservancy of the Rockies Fort Collins Colorado

**Keywords:** chestnut‐collared longspur, detection, dynamic, grassland, hierarchical habitat selection, lark bunting, multi‐scale, occupancy

## Abstract

Distribution models are increasingly being used to understand how landscape and climatic changes are affecting the processes driving spatial and temporal distributions of plants and animals. However, many modeling efforts ignore the dynamic processes that drive distributional patterns at different scales, which may result in misleading inference about the factors influencing species distributions. Current occupancy models allow estimation of occupancy at different scales and, separately, estimation of immigration and emigration. However, joint estimation of local extinction, colonization, and occupancy within a multi‐scale model is currently unpublished. We extended multi‐scale models to account for the dynamic processes governing species distributions, while concurrently modeling local‐scale availability. We fit the model to data for lark buntings and chestnut‐collared longspurs in the Great Plains, USA, collected under the Integrated Monitoring in Bird Conservation Regions program. We investigate how the amount of grassland and shrubland and annual vegetation conditions affect bird occupancy dynamics and local vegetation structure affects fine‐scale occupancy. Buntings were prevalent and longspurs rare in our study area, but both species were locally prevalent when present. Buntings colonized sites with preferred habitat configurations, longspurs colonized a wider range of landscape conditions, and site persistence of both was higher at sites with greener vegetation. Turnover rates were high for both species, quantifying the nomadic behavior of the species. Our model allows researchers to jointly investigate temporal dynamics of species distributions and hierarchical habitat use. Our results indicate that grassland birds respond to different covariates at landscape and local scales suggesting different conservation goals at each scale. High turnover rates of these species highlight the need to account for the dynamics of nomadic species, and our model can help inform how to coordinate management efforts to provide appropriate habitat configurations at the landscape scale and provide habitat targets for local managers.

## 1 INTRODUCTION

Species Distribution Models are increasingly being used to understand how landscape changes, such as habitat loss and fragmentation, and changes in the climate are affecting the processes driving the spatial and temporal distributions of plants and animals (Rushton, Ormerod, & Kerby, 2004). However, standard modeling frameworks often merely predict expected species distribution indices under future conditions based on established relationships between the patterns of species occurrence and vegetation or climatic characteristics while disregarding (a) the dynamic process that govern species distributions (MacKenzie, Nichols, Hines, Knutson, & Franklin, 2003) and (b) the hierarchical processes that drive habitat use at multiple scales (Cody, 1985). For example, distribution models often overlook (Gorzo et al., 2016) or make strong assumptions (Langham, Schuetz, Distler, Soykan, & Wilsey, 2015) about processes of local extinction and colonization that drive species persistence and range dynamics. Additionally, evaluating the relative importance of local and landscape features are important for biologically realistic species distributions and for predicting responses to habitat management and landscape conservation.

Models aimed at estimating species occurrence and occupancy dynamics while accounting for imperfect detection (MacKenzie et al., 2003) have been extended and applied to a wide variety of ecological questions and processes (see Bailey, MacKenzie, & Nichols, 2014 for a review). Standard dynamic occupancy models (MacKenzie et al., 2003) provide a framework to explicitly account for the processes driving patterns in species distributions rather than relying on the patterns alone. This approach provides a way to understand how occupancy distributions expand and contract and to investigate mechanistic hypotheses for occupancy dynamics over time. An understanding of these dynamics is important for making inference about how occupancy distributions may respond to climate and landscape change and avoids the unwise practice of inferring process from pattern (MacKenzie et al., 2018).

The more recent development of multi‐scale occupancy models allows estimation of occupancy probabilities at two scales (temporal or spatial; Nichols et al., 2008; Mordecai, Mattsson, Tzilkowski, & Cooper, 2011). Using spatial replication to estimate detection probabilities in standard occupancy models can result in biases in estimates of occupancy probabilities, due to the inability to separate whether the species was present at a survey location and not detected or the species was simply not present (Kendall & White, 2009). The multi‐scale model avoids this bias by estimating the probabilities that a species is present in the larger sample unit, available for detection at a survey location conditional on it being present at the coarser scale, and detected given it was present. This model was developed to account for the lack of independence among surveys (Hines et al., 2010; Nichols et al., 2008) but has since been applied to investigate the within‐season availability given the presence of a species during the breeding season (Mordecai et al., 2011) and the availability of species at local (e.g., territory) scales conditional on their presence on the landscape (Pavlacky, Blakesley, White, Hanni, & Lukacs, 2012).

The use of spatial subsamples extends the multi‐scale occupancy model in a way that allows for the evaluation of ecologically relevant hypotheses for hierarchical habitat use (Johnson, 1980). Scale is an important consideration in ecological studies, and the scales chosen for sampling and covariates should be appropriate for the species of interest and relevant to management (George & Zack, 2001; Wiens, 1989). For example, a species may not use a particular site because the local habitat may not be suitable, even if the landscape conditions are. Conversely, the local habitat may be suitable but the site is embedded within a landscape with an unsuitable habitat amount and/or configuration, precluding selection of the site at the coarser scale. Hines, Nichols, and Collazo (2014) extended the Markovian multi‐scale model to estimate site‐level extinction and colonization probabilities, but this model does not allow inference about hierarchical habitat use. Despite these advances and their usefulness in answering questions regarding habitat use at multiple scales, general multi‐scale models allowing for extinction and colonization of sites between sampling occasions have not been published, though a frequentist version is available in Program MARK (White & Burnham, 1999).

Nomadic species present the ideal situation for applying dynamic multi‐scale occupancy models. Populations of a migratory species with low site fidelity from year to year may appear to exhibit large annual variation when monitored with fixed survey locations and/or relatively small spatial coverage, when the species is stable but individuals are moving around on the landscape. Not explicitly accounting for these movements may result in a misunderstanding of the factors influencing nomadic species’ distributions and hinder conservation and management efforts.

One group of species showing nomadic tendencies is grassland birds (Cody, 1985; Igl & Johnson, 1999). Structural habitat characteristics of grasslands change rapidly because they are heavily influenced by local precipitation (Orians & Wittenberger, 1991) and disturbances, such as fire and grazing (Bragg & Steuter, 1996), and grassland birds must select breeding sites based on vegetation conditions present in the early spring (George, Fowler, Knight, & McEwen, 1992; Niemuth, Solberg, & Shaffer, 2008). Additionally, declines in grassland bird populations in North America have become a major conservation concern (Brennan & Kuvlesky, 2005; Peterjohn, 2003). Numerous conservation efforts have been implemented to counter these declines (Brennan & Kuvlesky, 2005), yet it is difficult to provide efficient and effective management without an understanding of the factors driving grassland bird distributions at the landscape and local levels. Here, we describe a dynamic, multi‐scale occupancy model and then apply the model to point count data collected in the Great Plains of the U.S. to identify the factors influencing landscape‐ and territory‐level occupancy and dynamics of two grassland bird species—lark bunting (*Calamospiza melanocorys*) and chestnut‐collared longspur (*Calcarius ornatus*). Finally, we discuss potential applications of dynamic, multi‐scale models for addressing ecological questions and informing management.

## 2 METHODS

### 2.1 Study area

**Figure 1:**
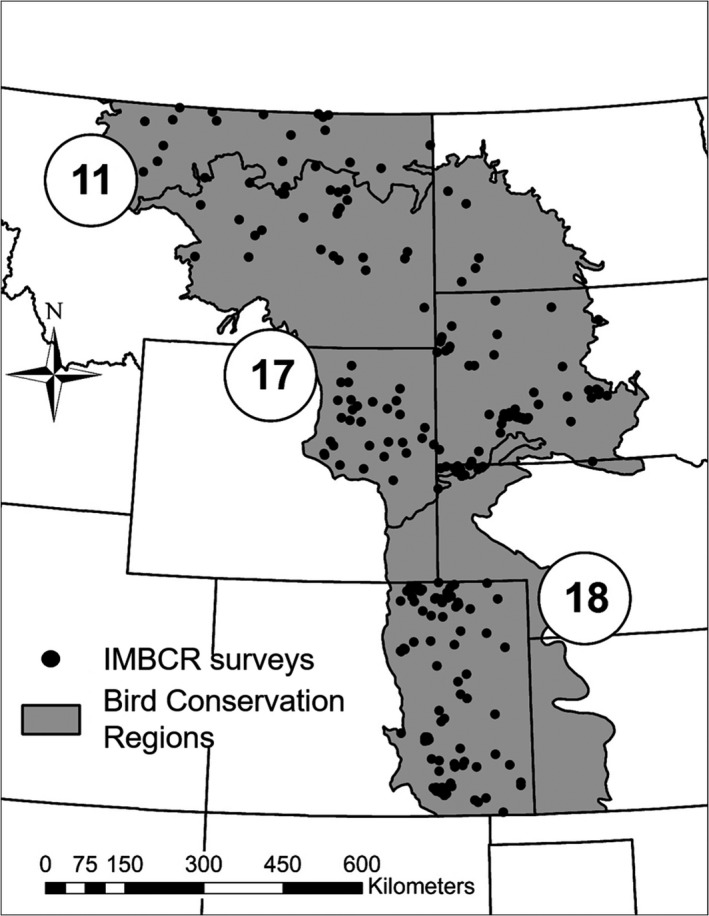
Location of 1‐km^2^ survey grids surveyed as part of the Integrated Monitoring in Bird Conservation Regions and used to investigate multi‐scale occupancy dynamics of grassland birds in the Great Plains, USA, 2010–2016. The study area included portions of the Prairie Potholes (11), Badlands and Prairies (17), and Shortgrass Prairie (18) Bird Conservation Regions

We used breeding season occurrence data collected in three Bird Conservation Regions (BCR; NABCI 2000) in the short‐ and mixed‐grass prairies of the western Great Plains of the U.S. covering approximately 652,000 km^2^ as part of the Integrated Monitoring in Bird Conservation Regions program (IMBCR; Pavlacky et al., 2017; Figure 1). The IMBCR program sampled the portion of the Prairie Potholes BCR within Montana, the entire Badlands and Prairies BCR, and the Shortgrass Prairie BCR north of the southern borders of Colorado and Nebraska.

### 2.2 Sampling frame

The IMBCR program employed a nested, stratified, probabilistic sampling design (Pavlacky et al., 2017). IMBCR strata were nested within the intersection of BCRs and states and were delineated using fixed attributes, such as land ownership boundaries, elevation zones, major river systems, and wilderness/roadless designations. The IMBCR sampling frame consisted of a uniform, 1‐km^2^ grid superimposed on each stratum. Grid cells were selected for sampling using Generalized Random‐Tessellation Stratification (Stevens & Olsen, 2004), and all grid cells within strata had a non‐zero and equal probability of selection. The design sampled vegetation in proportion to availability within strata, but we removed grid cells containing forest vegetation to facilitate the study of grassland‐specific occupancy in relation to spring green‐up, resulting in 252 unique grids in the final data set.

### 2.3 Field methods

Each 1‐km^2^ grid cell selected for a survey contained 16 point count stations spaced 250 m apart. Trained observers attempted to visit all 16 point count stations within a grid cell in a single morning, beginning approximately 30 min before sunrise and ending no later than five hours after official sunrise. The seasonal timing of point count surveys was dependent upon elevation, latitude, and habitat of grid cells to ensure that surveys were conducted when individuals had returned to breeding grounds and were actively defending territories (i.e., singing). Observers conducted 6‐min avian point count surveys at each point using standard distance sampling (Buckland et al., 2001) and removal in time (Alldredge, Pollock, Simons, Collazo, & Shriner, 2007) protocols. The initial detection of each individual or group of individuals of each species was recorded in 2‐min intervals, and distance to the individual or group was determined using a laser range finder (Pavlacky, Blakesley et al., 2012). To simplify interpretation of the availability parameter (θ), we truncated detections at 125 m resulting in independent 5‐ha point count plots with no overlap.

### 2.4 Model development

We expand upon the existing statistical theory of site occupancy models developed by MacKenzie and colleagues (MacKenzie et al., 2003, 2018) by combining dynamic, single‐scale models (MacKenzie et al., 2003) with static, multi‐scale models (Mordecai et al., 2011; Nichols et al., 2008; Pavlacky, Blakesley et al., 2012). Consider a sample design where *N* sample units are subsampled by *R* survey stations to determine the presence or absence of a species. In our example, the randomly selected 1‐km^2^ grid cells are the sampling units for which we estimated landscape‐scale occupancy. The occupancy status at each of the *R* survey stations within a sample unit can vary spatially. We considered the point count locations within each grid cell to be the survey stations and represent local‐scale occupancy. At each survey station, investigators conduct *K* repeated surveys using methodologies appropriate for detecting the species of interest. Repeat surveys at each survey station or a removal design can be used to estimate the probability of detecting the species (MacKenzie et al., 2018). In our application, we used a removal design to estimate detection probabilities, with 3 2‐min intervals as the repeat surveys (Pavlacky, Blakesley et al., 2012). The multi‐scale aspect of the model can be conceptualized as a within‐season robust design (Pollock, 1982) to estimate detection at *K* secondary occasions within *R* primary occasions and availability at *R* primary occasions within *T* tertiary survey occasions of the *N* sampling units (Nichols et al., 2008). The dynamic aspect of the model can be thought of as a between‐season robust design to estimate transitions between *T* tertiary survey occasions of the *N* sampling units, where surveys of the *N* sampling units conducted in subsequent *T* years are used to estimate dynamic parameters representing colonization and extinction of the species at the sample unit scale (MacKenzie et al., 2003). The model was initially developed for use with spatially nested sampling units but can also be applied to situations with a temporally nested sampling framework, as described in Mordecai et al. (2011).

We employed a sampling framework that exactly mirrors the IMBCR design with time occasions nested within point count plots, spatial subsamples nested within larger sampling units and sampling units nested within years. The dynamic multi‐scale occupancy model allows us to estimate (a) the probability a grid is occupied by a species in the first year of the study (initial occupancy, ψ), (b) the probability a grid is occupied by the species in year *t*, given the species was absent in *t*−1 (colonization, γ), (c) the probability a grid is not occupied by the species in year *t*, given the species was present in *t*−1 (extinction, ε), (d) the probability the species is present at a point count location, given it is present in the grid (θ), and (e) the probability the species is detected at a point count location, given it is present at the point count location (*p*).

We formulated the model as a state‐space model as in Mordecai et al. (2011) but modified it to allow for changes in grid‐level occupancy state across years. The state process submodel includes the latent states for occupancy at the grid and point count levels. The true occupancy state of grid *i* in year 1, $z_{i,1}$, is described by

$$z_{i,1}∼\text{Bern}\left(\psi_{i}\right)$$

for *i *=* *1, …, *N*. True grid‐level occupancy states in subsequent years are a function of the dynamic parameters

$$z_{i,t}∼\text{Bern}\left(\left(1-\varepsilon_{i,t-1}\right)\times z_{i,t-1}+\gamma_{i,t-1}\times \left(1-z_{i,t-1}\right)\right)$$

for *t *=* *2, …, *T*. The true occupancy state of a point count location is conditional upon the occupancy status of the corresponding grid and is described by

$$u_{i,j,t}∼\text{Bern}\left(\theta_{i,j,t}\times z_{i,t}\right)$$

for *j *=* *1, …, *R*. The observation submodel is conditional upon occupancy states at point counts and is denoted as

$$y_{i,j,k,t}∼\text{Bern}\left(p_{i,j,k,t}\times u_{i,j,t}\right)$$

for *k *=* *1, …, *K*, where $y_{i,j,k,t}$ indicates whether the species of interest was detected in survey *k* at point count *j* in grid *i* and year *t* (not detected = 0, detected = 1).

In addition to the primary parameters in the dynamic, multi‐scale occupancy model, other parameters may be of interest and can be derived from the primary parameters (Royle & Kéry, 2007). First, the occupancy probability at *t* can be calculated using the recursive equation

$$\psi_{t}=\psi_{t-1}\left(1-\varepsilon_{t-1}\right)+\left(1-\psi_{t-1}\right)\gamma_{t-1}$$

for *t *=* *2, …, *T*. The unconditional probability of local‐scale occupancy is calculated as

$$\delta_{t}=\psi_{t}\theta_{t}$$

for *t *=* *2, …,*T*. Site turnover is defined as the probability that a randomly chosen occupied site is newly occupied (Nichols, Boulinier, Hines, Pollock, & Sauer, 1998) and is calculated as

$$\tau_{t}=\frac{\gamma_{t-1}\left(1-\psi_{t-1}\right)}{\gamma_{t-1}\left(1-\psi_{t-1}\right)+\left(1-\varepsilon_{t-1}\right)\psi_{t-1}}$$

for *t *=* *2, …, *T*. Model parameters can be fit as a function of covariates as in other examples of state‐space occupancy models using a link function, such as the logit link (MacKenzie et al., 2003; Mordecai et al., 2011; Royle & Kéry, 2007).

### 2.5 Application: Grassland birds in the Northern Great Plains of the USA

We illustrate the utility of the model by developing hypotheses for hierarchical habitat use by the lark bunting and chestnut‐collared longspur (hereafter, bunting and longspur, respectively). Using expectations from the theory of hierarchical selection (Cody, 1985; Johnson, 1980), we assumed landscape relationships for the land cover of grassland and shrubland operated on the landscape‐scale occupancy of 1‐km^2^ grid cells and assumed local habitat relationships for canopy cover of grasses and shrubs operated on local‐scale occupancy (θ) of point count plots, conditional on the landscape‐scale occupancy state.

At the landscape‐scale, we expected bunting occupancy to be positively related to the amount of grassland and shrubland in the landscape, and expected longspur occupancy to be positively related to grassland cover but negatively related to shrubland cover. Likewise, we expected colonization to have positive relationships and extinction probabilities to have negative relationships to the same covariates (Dechant et al., 2002a,b). As birds returned to the breeding grounds in spring, we expected them to settle in areas with relatively robust vegetation growth and structure, reflected in the greenness of that vegetation (Ahlering, Johnson, & Faaborg, 2009). We hypothesized that the colonization of the bunting and longspur would increase with the extent of spring green‐up and local extinction of the species would decline with the extent of spring green‐up. Within a Geographic Information System (ESRI 2010), we calculated the proportion of grassland and shrub in each grid cell using the LANDFIRE existing vegetation type layer (USGS 2012). These layers were not available every year, so we used a fixed measure of these covariates from 2012 to represent landscape composition in each grid cell. To measure the annual variation in habitat conditions, we used the mean Normalized Difference Vegetation Index (NDVI; Didan & Huete, 2015) from May and June for each grid cell in each year. Because overall greenness varies from year to year, as well as spatially, in response to variation in precipitation and temperatures (Orians & Wittenberger, 1991), we standardized NDVI values for each year by subtracting the mean NDVI across all grids and dividing by the standard deviation. A standardized NDVI value of 0 represents the mean greenness for the year, positive values are greener than the mean, and negative values are less green than the mean. We modeled landscape‐scale occupancy, extinction, and colonization as functions of shrub cover, grass cover, and standardized NDVI measured at the grid.

At the local‐scale, we developed a series of hypotheses based on known local habitat relationships. Because the bunting prefers shrub‐dominated grasslands with intermediate grass height and high amounts of ground cover (Dechant et al., 2002b), we expected the local‐scale occupancy of buntings to increase with grass and shrub cover and be highest at intermediate grass heights. Longspurs typically use open grassland (Ribic et al., 2009) with shorter grass and large amounts of bare ground (Dechant et al., 2002a). We hypothesized that local‐scale occupancy of longspurs would be higher in areas with higher grass cover and lower shrub cover and grass height. We modeled local‐scale occupancy as a function of shrub cover, grass cover, and a quadratic effect of mean grass height measured at the point count location.

The covariates for local‐scale occupancy were based on vegetation measurements from IMBCR surveys. IMBCR observers recorded ocular measurements of the percent shrub and grass cover and mean grass height within 50 m of the center of each point count location. Percent cover measurements were binned into 0%, 1%, 5%, and 10% increments from 10% to 100%. As opposed to grassland and shrubland cover based on remotely‐sensed data, vegetation cover at the point level reflected the proportion of the ground covered by that vegetation type.

We used a logit link to model the parameters as functions of these covariates,

$$\text{logit}\left(\psi_{i}\right)=x_{i,1}\beta ,$$

$$\text{logit}\left(\varepsilon_{i,t}\right)=x_{i,t+1}\eta ,$$

$$\text{logit}\left(\gamma_{i,t}\right)=x_{i,t+1}\delta ,$$

and

$$\text{logit}\left(\theta_{i,t}\right)=w_{i,t+1}\alpha ,$$

where x and w are covariate matrices and β, η, δ, and α are regression coefficient vectors. We modeled separate detection probabilities for each year and used vague prior distributions for all estimate parameters:

$$p_{t}∼\text{Beta}\left(1,1\right)$$

and

$$\beta ,\eta ,\delta ,\alpha ∼\text{Normal}\left(0,100\right).$$

Fewer than 2% of point/year combinations were missing vegetation data. We interpolated values for those missing covariates by drawing missing values from an appropriate distribution (see Supporting Information Appendix S1 for more details). This approach to data interpolation incorporates the variation in observed covariate values into the interpolated values and, in turn, the coefficient estimates.

We estimated model parameters using Markov Chain Monte Carlo (MCMC) simulation implemented in JAGS 4.2.0 (Plummer, 2003, 2015) using the package R2jags in the R statistical computing environment (R Core Team 2015; Supporting Information Appendix S2). We obtained 25,000 MCMC samples and used a burn‐in period of 12,500 iterations.

## 3 RESULTS

**Figure 2:**
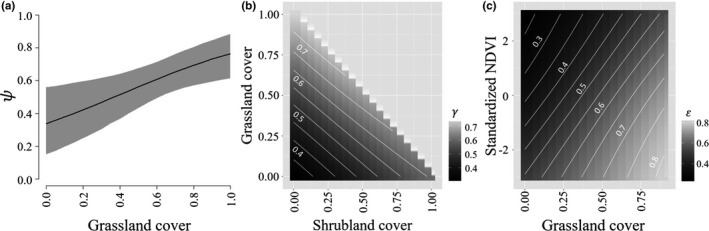
Relationships between covariates and landscape‐scale occupancy parameters for lark buntings in the Great Plains, USA, 2010–2016. (a) Initial occupancy probability (ψ) as a function of the proportion of 1‐km^2^ survey grids covered by grassland (95% credible intervals in gray). (b) Colonization probability (γ) as a function of the proportion of 1‐km^2^ survey grids covered by grassland and shrubland. (c) Extinction probability (ε) as a function of the proportion of 1‐km^2^ survey grids covered by grassland and standardized normalized differential vegetation index (NDVI), a measure of vegetation greenness. Estimates are shown at mean covariate values for covariates not shown in figure

**Figure 3:**
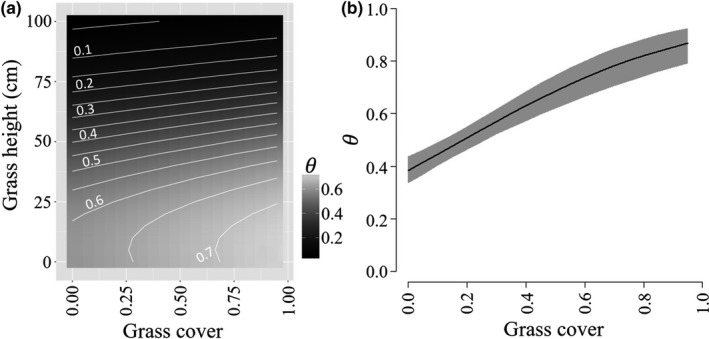
Relationships between (a) mean grass height and proportion grass cover at point count locations and local‐scale occupancy (θ) of lark buntings and (b) proportion grass cover at point count locations and θ for chestnut‐collared longspurs in the Great Plains, USA, 2010–2016. Gray shading represents 95% credible intervals

Initial occupancy of buntings at the grid level was strongly positively related to grassland cover (β = 1.98, 95% Credible Intervals [0.47, 3.43]; Supporting Information Table S1, Figure 2) but was not significantly influenced by the other covariates. Colonization of grids by buntings increased with shrubland (β = 4.62, [2.24, 7.38]) and grassland cover (β = 0.95, [0.12, 1.79]), and extinction of buntings decreased as grassland cover increased (β = −1.10, [−1.93, −0.21]) and for sites with higher than average NDVI (β = −0.71, [−1.09, −0.35]). Buntings responded to similar vegetation structure at the point count, with local‐scale occupancy increasing with grass cover (β = 0.57, [0.34, 0.81]; Supporting Information Table S1, Figure 3) and decreasing as a quadratic function of grass height (β = −0.0004, [−0.0007, −0.0001]). Detection of buntings was high and ranged from 0.76 [0.70, 0.81] in 2010 to 0.87 [0.83, 0.90] in 2016 (Supporting Information Table S2).

**Figure 4:**
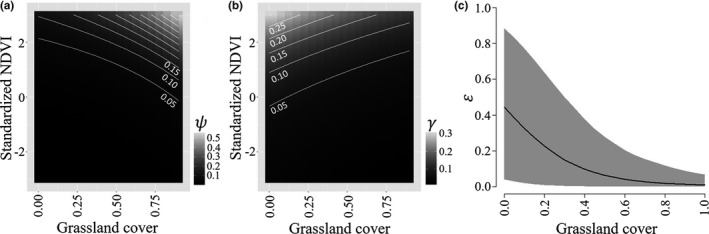
Relationships between covariates and landscape‐scale occupancy parameters for chestnut‐collared longspurs in the Great Plains, USA, 2010–2016. (a) Initial occupancy probability (ψ) and (b) colonization probability (γ) as a function of the proportion of 1‐km^2^ survey grids covered by grassland and the standardized normalized differential vegetation index (NDVI), a measure of vegetation greenness (95% credible intervals in gray). (c) Extinction probability (ε) as a function of the proportion of 1‐km^2^ survey grids covered by grassland and the standardized NDVI. Estimates are shown at mean covariate values for covariates not shown in figure

Occupancy of longspurs at grids in the first year was higher in grids with more grassland cover (β = 4.02, [0.05, 9.50]) and higher than average NDVI (β = 1.05, [0.39, 1.88]; Supporting Information Table S3, Figure 4), extinction decreased with increasing grassland cover (β = −6.43, [−13.52, −2.03]), and colonization decreased with increasing grassland cover (β = −1.51, [−3.10, −0.06]) and was higher at sites with higher NDVI values (β = 0.63, [0.09, 1.16]). Local‐level occupancy of longspurs was driven by grass cover (β = 2.54, [1.81, 3.32]; Supporting Information Table S3, Figure 3). Longspur detection probabilities were high in 2010 (*p *=* *0.89 [0.81, 0.95]) and declined across the study period (*p *=* *0.53 [0.25, 0.81] in 2016; Supporting Information Table S2).

**Figure 5:**
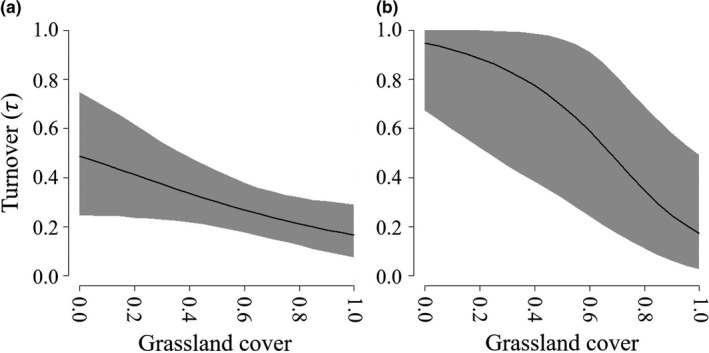
Turnover probabilities (τ) as a function of the proportion of 1‐km^2^ survey grids covered by grassland for (a) lark buntings and (b) chestnut‐collared longspurs in the Great Plains, USA, 2010–2016. Gray shading represents 95% credible intervals

Buntings were widely distributed on the landscape with high and increasing landscape‐scale occupancy probabilities (ψ) and were similarly distributed at the local scale (θ; Supporting Information Table S2). Approximately 47% of points were occupied (δ) by buntings in a given year. Longspurs were rare on the landscape within our study area, were locally prevalent in occupied grids, and approximately 4% of points were occupied in any given year. Turnover rates (τ) were high for the bunting with approximately 18% of grids newly occupied in any given year and even higher and more variable for the longspur, ranging from 0.15 to 0.53 (Supporting Information Table S2; Figure 5).

## 4 DISCUSSION

Understanding the processes driving the regional distribution of animals is important for evaluating biologically realistic hypotheses in conservation science and may be useful for informing the management of these populations. Using patterns to infer process involves retrospective speculation about the underlying population processes and the risk of erroneous conclusions may far outweigh the any benefits for species of high conservation concern (Martin, Kitchens, & Hines, 2007). Development of site occupancy models (Bailey et al., 2014; MacKenzie et al., 2003, 2018) have allowed investigators to explicitly estimate dynamic occupancy parameters to better understand the processes driving occupancy distributions. We combined dynamic and multi‐scale occupancy models to estimate these dynamic parameters and investigate hierarchical habitat selection by species. Our application of the model to two grassland bird species highlights the need to account for the nomadic nature of some species and provides information useful for the successful conservation of these species.

### 4.1 Dynamic multi‐scale occupancy model

Dynamic parameters can provide insight into the mechanisms driving a species’ distribution. For example, year‐specific occupancy estimates may be consistent from year to year but sites have high turnover rates. Conducting a distributional analysis using a single‐season model, one might infer that occupancy at a given site is stable, but as evidenced in our example, turnover rates could be very high but occupancy remains stable. If nomadic behavior sometimes results in suboptimal habitat use (Battin, 2004), then accounting for dynamics may improve parameter estimates for important habitat features, such as grassland patch size (Ribic et al., 2009). Another generality that is often made based on the response of density or occupancy to habitat conditions is that increased density or occupancy probabilities are indicative of “good” habitat. Van Horne (1983) cautions against this interpretation because sites with high abundance may be population sinks with low reproduction and/or survival. According to Van Horne (1983), it is better to define “good” habitat based on the response of vital rates. The dynamic occupancy model provides a means to model explicitly the vital rates (i.e., colonization and extinction) as a function of habitat variables.

Multi‐scale occupancy models can be used to test hypotheses relating to hierarchical habitat use, while accounting for smaller site selection being conditional on higher levels. Studies examining occupancy at only one scale can miss the influence of conditions at other scales on the presence of the species, resulting in biased habitat or landscape relationships. For example using the IMBCR design, habitat conditions may be suitable at the grid but not at a point count location. If an investigator only estimated occupancy at the grid, misleading conclusions could be drawn about the suitability of the habitat conditions for the species. Conversely, vegetation structure may be ideal at a point count location, but if it is nested within a matrix of unsuitable habitat, it is not likely to be occupied.

Occupancy is often used as an indicator of a species’ population status (Adams et al., 2013; Bart & Klosiewski, 1989; Davidson, 2004; Zielinski, Baldwin, Truex, Tucker, & Flebbe, 2013) because occupancy surveys are often easier and more cost effective than those used to measure abundance. However, occupancy may not be an adequate measure of abundance, and population declines may be occurring even though occupancy rates are stable (Noon, Bailey, Sisk, & McKelvey, 2012). An exception to this is if the sampling unit corresponds to an individual's home range, as with tigers (*Panthera tigris*; Hines et al., 2010), or a breeding pair's territory, as with spotted owls (*Strix occidentalis*; Azuma, Baldwin, & Noon, 1990; MacKenzie et al., 2003) and marbled murrelets (*Brachyramphus marmoratus*; Stauffer, Ralph, & Miller, 2004). The multi‐scale occupancy model can be used to approximate abundance by setting the area of local‐scale survey stations to approximate the home range or territory size of the species of interest. These can then be nested within a larger sampling unit to estimate distributional patterns of the species. Comparing local‐ and landscape‐scale occupancy estimates may identify declines in abundance before they begin to influence distributional patterns.

Past applications of the multi‐scale occupancy model have investigated species use and availability at two spatial or temporal scales (Mordecai et al., 2011; Nichols et al., 2008), but no general approach was available to explicitly model the processes influencing those patterns. Our dynamic multi‐scale model allows researchers to examine occupancy dynamics at the landscape‐scale and species distributions across nested scales in the same model. The model could be easily extended to community modeling or to account for misidentification or heterogeneity due to differences in abundance (Bailey et al., 2014). Additional hierarchical levels could also be included to model occupancy at coarser or finer spatial or temporal scales. Applications of this model can also help determine the relative importance of local and landscape processes shaping the occupancy distribution of species, which is not possible with existing Species Distribution Models. Researchers and managers can use this model to answer questions, such as: (a) what is the relative influence of local habitat conditions versus landscape habitat loss and fragmentation; and (b) how do we allocate resources to landscape conservation versus local habitat management to maximize the occupancy of the species? Insights into these types of questions can help inform how to coordinate management and conservation efforts to provide appropriate habitat configurations at the landscape scale and provide habitat targets for local managers.

### 4.2 Grassland birds in the Northern Great Plains

Our analysis focused on two grassland bird species with different habitat preferences and population statuses, but we found that both exhibited similar occupancy dynamics in response to varying habitat conditions. The bunting colonized sites with greater grassland and shrubland cover with higher probabilities (Supporting Information Table S1, Figure 2). These relationships suggest that buntings cued in on the amount of preferred habitat when selecting landscapes in which to breed, suggesting that intact shrublands may be particularly important for colonization by buntings when conditions are poor. Longspurs cued in on vegetation greenness, potentially selecting more agricultural areas especially during dry years. Responding primarily to vegetation greenness may result in high colonization rates in landscapes impacted by habitat loss where survival and productivity are lower, contributing to non‐optimal habitat use (Battin, 2004) and range contraction (Pavlacky, Possingham et al., 2012). Once occupancy during the breeding season is established at a site, both species were more likely to persist when grassland cover was higher and, for buntings, when vegetation was greener.

Our analysis included habitat variables we thought might be most important to the occupancy dynamics of grassland birds at landscape and local scales. However, we acknowledge that grassland birds may respond to other habitat features at both scales. Many studies conflate habitat loss and fragmentation, but species respond differently to each (Conner & Rudolph, 1991; Fischer & Lindenmayer, 2007; Lehtinen, Galatowitsch, & Tester, 1999). In dynamic landscapes, such as grasslands, and with nomadic species, such as grassland‐obligate birds, accounting for the amount and configuration of habitat on the landscape may be crucial for the conservation of these species (Fischer & Lindenmayer, 2007; Runge, Martin, Possingham, Willis, & Fuller, 2014). The multi‐scale model provides an opportunity to test hypotheses about how habitat loss and fragmentation influence dynamic parameters and predict changes in distributions before they are seen, all while modeling influences at a finer scale and accounting for detection. We chose to use NDVI during the breeding season as a measure of vegetation condition; however, there is justification for using a number of metrics (e.g., precipitation, greenness indices, Palmer Drought Severity Index) and time periods (Ahlering et al., 2009; Gorzo et al., 2016; Lipsey & Naugle, 2017). Teasing apart the differences in these metrics is beyond the scope of this paper, but information on how weather affects demographic parameters can be combined with occupancy data within an integrated population model (Abadi, Giminez, Ullrich, Arlettaz, & Schaub, 2010; Hostetler, Sillett, & Marra, 2015) to refine our understanding of the mechanics driving species distributions and improve parameter estimation.

The response of buntings and longspurs to annual changes in vegetation conditions suggest that it is important to provide habitat across coarse spatial scales to allow for annual variation in vegetation conditions (Hanski, 1998). Grids with complete grassland coverage had the lowest turnover rates (Figure 5), suggesting that large areas of intact habitat may serve as refugia. However, turnover was still very high (Supporting Information Table S2), and large blocks of grassland should be maintained across bunting and longspur ranges to provide refugia to buffer against the effects of local environmental variation. Both grassland bird species chose similar habitat metrics at coarse and fine scales. The amount of grassland covering a grid was a significant influence on all landscape‐scale occupancy parameters (i.e., ψ, γ, and ε) for buntings and longspurs. At the point count scale, both species also responded positively to increased grass cover. This result may be intuitive since these birds have evolved in the grasslands of North America, but it highlights that these populations benefit from large blocks of grassland habitat and provides management targets for the preferred vegetation structure at the territory scale.

Patterns in landscape‐ and local‐scale occupancy of the two species reflected patterns in regional abundance. We did not observe a decoupling of landscape‐ and local‐scale occupancy for buntings, which have had stable populations in the Great Plains over the study period (Woiderski et al., 2018). Conversely, local‐scale occupancy probabilities of longspurs declined over the study period, coinciding with sharp population declines in the region (Woiderski et al., 2018), while landscape‐scale occupancy increased slightly. In this case, local‐scale occupancy provides a measure of prevalence measured by the fraction of point plots occupied when grids are occupied (Pavlacky, Blakesley et al., 2012), but the area around each point count (4.9 ha) was large enough to contain breeding territories of several pairs of either species (bunting: 0.2–1.1 ha, Dechant et al., 2002b; longspur: 0.2–1.0 ha; Dechant et al., 2002a). The decline in local‐scale occupancy may serve as early warning of an extinction debt (Tilman, May, Lehman, & Nowak, 1994) as a consequence of the apparent non‐optimal habitat use (Battin, 2004) mentioned above.

Landscape‐scale monitoring programs may be necessary to provide context for dynamics of nomadic species. The IMBCR program survey area covered approximately 1.8 million km^2^ in 2016 encompassing much of the western Great Plains and Intermountain West in the U.S. (Woiderski et al., 2018), allowing us to sample across a wide range of vegetation conditions and giving us a complete picture of the dynamics of grassland bird populations. If survey areas are small, compared to the movements of the species of interest, occupancy may fluctuate widely across years, implying large swings in populations, when distributions may have just shifted outside of the study area (George et al., 1992; Niemuth et al., 2008). Nomadic species may function as metapopulations across coarse scales, such as BCRs, with the overall population being maintained by local extinction and recolonization events (Hanski, 1998; Lande, 1988; Levins, 1969, 1970), and they would likely benefit from consideration of landscape‐level habitat configurations and coordinated management efforts (Runge et al., 2014; Wiens, 1995).

## CONFLICT OF INTEREST

The authors have no conflicts of interest to report.

## AUTHOR CONTRIBUTIONS

The study was conceptualized by A.W.G., D.C.P., and T.L.G., and A.W.G. performed the analysis and led the writing of the manuscript. All authors contributed critically to the drafts and gave final approval for publication.

## Data Availability

All IMBCR data are available from Bird Conservancy of the Rockies upon request.
