# Supplementary material for: A dynamic multi‐scale occupancy model to estimate temporal dynamics and hierarchical habitat use for nomadic species
Source: Ecol Evol. 2019 Feb 5;9(2):793–803. doi: 10.1002/ece3.4822 (PMC6362800; doi:10.1002/ece3.4822)
Supplement: Supplementary file 1 [file ECE3-9-793-s001.docx]

**Appendix S1**. Description of covariate interpolation and estimates of main parameters and covariate coefficients for Lark Bunting and Chestnut-collared Longspur from dynamic multi-scale occupancy model

*Covariate interpolation*

We interpolated covariate values for the few point/year combinations where vegetation were not collected or had errors. We assumed that shrub cover (sc) and grass cover (gc) were distributed as

$$w_{i,j,t}^{sc}\sim Beta(\alpha_{sc},\beta_{sc})$$

and

$w_{i,j,t}^{gc}\sim Beta(\alpha_{gc},\beta_{gc})$,

and grass height (gh) was distributed as

$w_{i,j,t}^{gh}\sim Gamma(\frac{\mu_{gh}^{2}}{\sigma_{gh}^{2}},\frac{\mu_{gh}}{\sigma_{gh}^{2}})$,

where $\mu_{gh}$ and $\sigma_{gh}^{2}$ were the mean and variance of grass height, respectively. We assumed vague priors for all shape parameters

$\alpha_{sc},\beta_{sc},\alpha_{gc},\beta_{gc}\sim dunif(0,500)$,

$\mu_{gh}\sim dunif(0,200)$,

and

$\sigma_{gh}\sim dunif(0,50)$.

Collected data helped inform shape parameters for these distributions, and missing values were randomly drawn from these distributions in each MCMC iteration.

**Table S1.** Mean, standard deviation (SD), and 95% credible intervals (CrI) for estimates of covariate coefficients explaining variation in dynamic multi-scale occupancy parameters of lark buntings in the Great Plains, USA, 2010-2016. Parameters include initial occupancy ($\psi$), colonization ($\gamma$), and extinction (𝜀) at the large scale and small-scale occupancy ($\theta$).Coefficients with 95% CrIs not including 0 are shown in bold.

| Covariate | Mean | SD | 95% CrI |
| --- | --- | --- | --- |
| $\psi$ |  |  |  |
| Intercept | -0.855 | 0.556 | (-1.965, 0.232) |
| Shrubland cover^1^ | 1.410 | 1.261 | (-0.957, 3.968) |
| **Grassland cover**^2^ | **1.934** | **0.755** | **(0.465, 3.429)** |
| Standardized NDVI^3^ | -0.245 | 0.286 | (-0.813, 0.320) |
| $\gamma$ |  |  |  |
| **Intercept** | **-1.179** | **0.309** | **(-1.802, -0.590)** |
| **Shrubland cover** | **4.617** | **1.335** | **(2.238, 7.375)** |
| **Grassland cover** | **0.953** | **0.426** | **(0.116, 1.792)** |
| Standardized NDVI | -0.179 | 0.154 | (-0.493, 0.111) |
| 𝜀 |  |  |  |
| **Intercept** | **-0.778** | **0.332** | **(-1.447, -0.173)** |
| Shrubland cover | -0.843 | 0.711 | (-2.238, 0.502) |
| **Grassland cover** | **-1.096** | **0.430** | **(-1.926, -0.211)** |
| **Standardized NDVI** | **-0.711** | **0.190** | **(-1.094, -0.349)** |
| $\theta$ |  |  |  |
| **Intercept** | **0.470** | **0.083** | **(0.310, 0.635)** |
| Shrub cover^4^ | -0.217 | 0.390 | (-1.003, 0.538) |
| **Grass cover**^5^ | **0.569** | **0.122** | **(0.338, 0.814)** |
| Grass height | 0.004 | 0.007 | (-0.010, 0.019) |
| **Grass height quadratic** | **-0.0004** | **0.0001** | **(-0.0007, -0.0001)** |

^1^ Proportion of 1-km^2^ sampling grid covered in shrubland

^2^ Proportion of 1-km^2^ sampling grid covered in grassland

^3^ Normalized Difference Vegetation Index (NDVI) from May-June standardized to have a mean of 0 and standard deviation of 1

^4^ Percent ground cover within 50 m of point count center covered by shrubs

^5^ Percent ground cover within 50 m of point count center covered by grass

**Table S2.** Mean derived parameter estimates for lark buntings and chestnut-collared longspurs in the Great Plains, USA, 2010-2016. Parameters include large-scale occupancy probability ($\psi$), small-scale occupancy probability conditional on presence at the large-scale ($\theta$), unconditional small-scale occupancy ($\delta=\psi\theta$), turnover probability ($\tau$), and detection probability (*p*). All estimates except *p* were estimated at mean covariate values, and 95% credible intervals are shown in parentheses.

| Year | $\psi$ | $\theta$ | $\delta$ | $\tau$ | *p* |
| --- | --- | --- | --- | --- | --- |
| Lark bunting |  |  |  |  |  |
| 2010 | 0.634 (0.520, 0.741) | 0.671 (0.650, 0.691) | 0.425 (0.348, 0.499) | - | 0.761 (0.705, 0.810) |
| 2011 | 0.717 (0.661, 0.768) | 0.663 (0.645, 0.681) | 0.475 (0.437, 0.511) | 0.255 (0.165, 0.357) | 0.817 (0.781, 0.850) |
| 2012 | 0.745 (0.695, 0.793) | 0.645 (0.630, 0.659) | 0.480 (0.446, 0.513) | 0.188 (0.150, 0.228) | 0.826 (0.789, 0.861) |
| 2013 | 0.755 (0.703, 0.804) | 0.635 (0.620, 0.650) | 0.480 (0.445, 0.513) | 0.167 (0.133, 0.206) | 0.802 (0.766, 0.835) |
| 2014 | 0.758 (0.705, 0.808) | 0.629 (0.611, 0.645) | 0.477 (0.441, 0.511) | 0.160 (0.124, 0.201) | 0.823 (0.792, 0.851) |
| 2015 | 0.760 (0.706, 0.81) | 0.634 (0.618, 0.649) | 0.481 (0.445, 0.516) | 0.158 (0.119, 0.200) | 0.844 (0.819, 0.868) |
| 2016 | 0.760 (0.706, 0.811) | 0.626 (0.607, 0.644) | 0.476 (0.440, 0.511) | 0.157 (0.117, 0.200) | 0.867 (0.829, 0.898) |
| Chestnut-collared longspur |  |  |  |  |  |
| 2010 | 0.023 (0.004, 0.061) | 0.689 (0.623, 0.754) | 0.016 (0.003, 0.042) | - | 0.890 (0.808, 0.950) |
| 2011 | 0.044 (0.023, 0.078) | 0.646 (0.585, 0.706) | 0.029 (0.015, 0.051) | 0.529 (0.623, 0.864) | 0.874 (0.782, 0.940) |
| 2012 | 0.064 (0.036, 0.100) | 0.525 (0.476, 0.574) | 0.034 (0.019, 0.053) | 0.340 (0.585, 0.469) | 0.766 (0.679, 0.842) |
| 2013 | 0.083 (0.048, 0.125) | 0.461 (0.416, 0.506) | 0.038 (0.022, 0.058) | 0.254 (0.476, 0.337) | 0.373 (0.139, 0.895) |
| 2014 | 0.101 (0.057, 0.150) | 0.440 (0.392, 0.488) | 0.044 (0.025, 0.067) | 0.204 (0.416, 0.281) | 0.717 (0.596, 0.813) |
| 2015 | 0.118 (0.066, 0.176) | 0.470 (0.422, 0.518) | 0.055 (0.031, 0.084) | 0.172 (0.392, 0.250) | 0.620 (0.526, 0.708) |
| 2016 | 0.134 (0.074, 0.203) | 0.443 (0.392, 0.493) | 0.059 (0.032, 0.091) | 0.149 (0.422, 0.230) | 0.528 (0.251, 0.813) |

**Table S3.** Mean, standard deviation (SD), and 95% credible intervals (CrI) for estimates of covariate coefficients explaining variation in dynamic multi-scale occupancy parameters of chestnut-collared longspurs in the Great Plains, USA, 2010-2016. Parameters include initial occupancy ($\psi$), colonization ($\gamma$), and extinction (𝜀) at the large scale and small-scale occupancy ($\theta$).Coefficients with 95% CrIs not including 0 are shown in bold.

| Covariate | Mean | SD | 95% CrI |
| --- | --- | --- | --- |
| $\psi$ |  |  |  |
| **Intercept** | **-6.460** | **2.314** | **(-11.674, -2.800)** |
| Shrubland cover^1^ | -0.974 | 4.258 | (-9.863, 7.157) |
| **Grassland cover**^2^ | **4.017** | **2.434** | **(0.049, 9.497)** |
| **Standardized NDVI**^3^ | **1.054** | **0.377** | **(0.387, 1.881)** |
| $\gamma$ |  |  |  |
| **Intercept** | **-2.823** | **0.460** | **(-3.770, -1.967)** |
| Shrubland cover | -0.020 | 1.109 | (-2.388, 1.989) |
| **Grassland cover** | **-1.512** | **0.761** | **(-3.095, -0.064)** |
| **Standardized NDVI** | **0.631** | **0.275** | **(0.086, 1.158)** |
| $\varepsilon$ |  |  |  |
| Intercept | -0.220 | 1.301 | (-3.047, 2.145) |
| Shrubland cover | -1.134 | 3.470 | (-8.751, 4.947) |
| **Grassland cover** | **-6.426** | **3.058** | **(-13.524, -2.035)** |
| Standardized NDVI | 0.875 | 1.010 | (-0.816, 3.116) |
| $\theta$ |  |  |  |
| **Intercept** | **-0.607** | **0.215** | **(-1.026, -0.177)** |
| Shrub cover^4^ | -0.183 | 1.575 | (-3.254, 2.899) |
| **Grass cover**^5^ | **2.544** | **0.382** | **(1.814, 3.323)** |
| Grass height | 0.004 | 0.018 | (-0.034, 0.039) |
| Grass height quadratic | 0.00006 | 0.00036 | (-0.00059, 0.00082) |

^1^ Proportion of 1-km^2^ sampling grid covered in shrubland

^2^ Proportion of 1-km^2^ sampling grid covered in grassland

^3^ Normalized Difference Vegetation Index (NDVI) from May-June standardized to have a mean of 0 and standard deviation of 1

^4^ Percent ground cover within 50 m of point count center covered by shrubs

^5^ Percent ground cover within 50 m of point count center covered by grass
