# Supplementary material for: A dynamic multi‐scale occupancy model to estimate temporal dynamics and hierarchical habitat use for nomadic species
Source: Ecol Evol. 2019 Feb 5;9(2):793–803. doi: 10.1002/ece3.4822 (PMC6362800; doi:10.1002/ece3.4822)
Supplement: Supplementary file 2 [file ECE3-9-793-s002.docx]

**Appendix S2.** Dynamic multi-scale occupancy model JAGS code

model{

# Priors

for(t in 1:T) { # year-specific detection

p[t]~dbeta(1,1)

}

for(i in 1:4) { # initial occupancy coefficients

beta[i]~dnorm(0,0.01)

}

for(i in 1:4) {

eta[i]~dnorm(0,0.01) # extinction coefficients

delta[i]~dnorm(0,0.01) # colonization coefficients

}

for(i in 1:5) {

alpha[i]~dnorm(0,0.01) # theta coefficients

}

# Interpolate missing covariate values

for(t in 1:T) {

pt.shrub.cov.a[t]~dunif(0,500)

pt.shrub.cov.b[t]~dunif(0,500)

pt.grass.cov.a[t]~dunif(0,500)

pt.grass.cov.b[t]~dunif(0,500)

pt.grass.ht.mn[t]~dunif(0,200)

pt.grass.ht.sd[t]~dunif(0,50)

pt.grass.ht.var[t]<-pow(pt.grass.ht.sd[t],2)

}

for(i in 1:n.grid) {

for(j in 1:n.pt.grid) {

for(t in 1:T) {

pt.shrub.cov[i,j,t]~dbeta(pt.shrub.cov.a[t],pt.shrub.cov.b[t]) # point-level shrub cover

pt.grass.cov[i,j,t]~dbeta(pt.grass.cov.a[t],pt.grass.cov.b[t]) # point-level grass cover

pt.grass.ht[i,j,t]~dgamma(pow(pt.grass.ht.mn[t],2)/pt.grass.ht.var[t],pt.grass.ht.mn[t]/pt.grass.ht.var[t]) # point-level grass height

}

}

}

# Sample large-scale occupancy parameters

for(i in 1:n.grid) {

logit(psi[i])<-beta[1] + beta[2]*grid.shrub.cov[i] + beta[3]*grid.grass.cov[i] + beta[4]*grid.ndvi.std[i,1]

z.grid[i,1]~dbern(psi[i]) # Sample large-scale occupancy, year 1

for(t in 2:T) {

logit(eps[i,t-1])<-eta[1] + eta[2]*grid.shrub.cov[i] + eta[3]*grid.grass.cov[i] + eta[4]*grid.ndvi.std[i,t] # Calculate grid-level extinction

logit(gamma[i,t-1])<-delta[1] + delta[2]*grid.shrub.cov[i] + delta[3]*grid.grass.cov[i] + delta[4]*grid.ndvi.std[i,t] # Calculate grid-level colonization

z.grid[i,t]~dbern(z.grid[i,t-1]*(1-eps[i,t-1]) + (1-z.grid[i,t-1])*gamma[i,t-1]) # sample large-scale occupancy, year >1

tau.z.hold[i,t] <- (1-z.grid[i,t-1])*z.grid[i,t] # Used to calculate turnover

}

# Sample small-scale occupancy and detection

for(t in 1:T) {

for(j in 1:n.pt.grid) { # loop through the number of points in each grid

logit(theta[i,j,t])<-alpha[1] + alpha[2]*pt.shrub.cov[i,j,t] + alpha[3]*pt.grass.cov[i,j,t] + alpha[4]*pt.grass.ht[i,j,t] +alpha[5]*pow(pt.grass.ht[i,j,t],2) # Calculate theta

u[i,j,t] ~ dbern(theta[i,j,t]) # sample point-level occupancy

y[i,j,t] ~ dbinom(p[t]*u[i,j,t], J[i,j,t]) # Detection process

}

}

}

# Posterior predictive distributions

for(t in 2:T) { # Calculate turnover

tau.finite[t] <- sum(tau.z.hold[,t])/sum(z.grid[,t])

}

for(t in 1:T) { # Calculate finite occupancy rates

psi.finite[t]<-mean(z.grid[,t])

theta.finite[t]<-mean(u[,,t])

}

}
